# SPINK1 contributes to proliferation and clonal formation of HT29 cells through Beclin1 associated enhanced autophagy

**DOI:** 10.32604/or.2022.027058

**Published:** 2023-01-05

**Authors:** NA HU, SHIQING ZHANG, AQUAN JIN, LIANYING GUO, ZHENYUN QU, JUN WANG

**Affiliations:** 1 Department of Pathology, Northwest Women’s and Children’s Hospital, Xi’an, 710061, China; 2 Department of Pathophysiology, College of Basic Medical Sciences, Dalian Medical University, Dalian, 116000, China; 3 Department of Respiratory and Critical Care Medicine, Guizhou Provincial People’s Hospital, Guiyang, 550000, China

**Keywords:** SPINK1, Colorectal carcinoma, Autophagy, Beclin1

## Abstract

We aimed to explore the molecular mechanism that were involved in SPINK1-induced proliferation and clonogenic survival of human colorectal carcinoma (CRC) HT29 cells. Initially, we generated HT29 cells either permanently silencing or overexpressing SPINK1 protein. The results showed that SPINK1 overexpression (OE) significantly stimulated the proliferation and clonal formation of HT29 cells at the varied time points. Secondly, we found SPINK1 OE enhanced the ratio of LC3II/LC3I and the level of autophagy-related gene 5 (ATG5), whereas SPINK1 knockdown (Kd) reversed the above outcome under normal culturing and/or fasting condition in the cells, indicating its role in autophagy enhancement. Moreover, LC3-GFP-transfected SPINK1-OE HT29 cells strengthened the fluorescence intensity compared with the untransfected control. Chloroquine (CQ) significantly decreased the level of autophagy in both control and SPINK1-OE HT29 cells. The autophagy inhibitors, CQ and 3-Methyladenine (3-MA), remarkably inhibited the proliferation and colony formation of SPINK1-OE HT29 cells, while ATG5 upregulation resulted in the growth of the cells, suggesting the important function of autophagy in cell’s growth. Thirdly, SPINK1-induced autophagy was independently of mTOR signaling as p-RPS6 and p-4EBP1 were activated in SPINK1-OE HT29 cells. Instead, Beclin1 up and down regulation were clearly observed in SPINK1-OE and SPINK1 Kd HT29 cells, respectively. Moreover, Beclin1 silencing apparently reduced autophagy in SPINK1-OE HT29 cells, indicating that SPINK1-induced autophagy was closely associated with Beclin1. Collectively, SPINK1-promoted proliferation and clonal formation of HT29 cells were closely associated with Beclin1 associated enhanced autophagy. The above findings would open a new window for probing the role of SPINK1-related autophagic signaling in the pathogenesis of CRC.

## Introduction

Human serine protease inhibitor Kazal type 1 (SPINK1) is also known as tumor associated trypsin inhibitor (TATI), however, the precise molecular mechanisms underlying its oncogenic action remains largely unknown and controversial [1] SPINK1 has been found to be overexpressed in multiple types of tumors [2], and its overexpression was closely linked to the proliferation and invasion of several kinds of tumor cells [2,3] and the high level of its expression was associated with disease progression and poor prognosis in certain types of cancer patients [4]. For the past decade, it has drawn much attention that Spink3 (serine protease inhibitor Kazal type 3, human homolog of SPINK1) was reported to be an autophagy negative regulator since the deletion of this gene resulted in increased microtubule-associated protein 1 light chain 3 (LC3) and excessive autophagy in pancreatic acinar cells of Spink3 knockout mice [5]. It is known that autophagy is a highly conserved cellular self-eating process, in which damaged or superfluous proteins and organelles are engulfed by double-membrane autophagosomes and degraded by lysosomal enzymes [6]. In addition to its primary role in stress, autophagy is also involved in the development of cancer [7]. Though the exact roles of autophagy played in cancers remains largely unknown, its dual functions in the pathogenesis of tumor growth have been noticed [7]. However, the links between SPINK1 and autophagic process in cancer cell growth remain undiscovered [8], as well as the underlying molecular mechanisms with which SPINK1 may contribute to tumor development. Thus, we hypothesized that SPINK1 may possibly exert its carcinogenic effects on tumor cell’s survival at least in part via autophagy signaling and regulation.

To understand the relationships among SPINK1, autophagy, and tumor growth, we chose human colorectal cancer (CRC) HT29 cells as the typical objects to be studied. It is known that SPINK1 was overexpressed in some types of colon adenocarcinoma and it promotes CRC progression in chickembryo study [9].

But the underlying mechanisms remains unclear [1–3]. In this study, we came across an autophagic molecular event with which SPINK1 increased the proliferation and clonogenic survival of human embryonic kidney (HEK) 293T cells. We demonstrated that the above event was closely associated with Beclin-1 regulated, enhanced autophagy. These findings may open a new window for the understanding of the signaling pathways via which SPINK1 may act as TATI [2,10].

## Materials and Methods

### Cell culture, vectors, antibodies and reagents

As a gift, HT29 cells was provided by Prof. Penghui Ma (Dalian Medical University, China) while human embryonic kidney (HEK) 293T cell line was provided by Prof. Zeyao Tang (Dalian Medical University, China), and these two cell lines were coming indirectly from American Type Culture Collection (ATCC), and AsPC-1 cells was obtained from Prof. Yamamura K (Kumamoto University, Japan). All cell lines were grown either in McCoy’s (Sangon Biotech Co., Ltd., Shanghai, China), RPMI-1640 or DMEM medium (Sangon Biotech, Shanghai, China) with 10% fetal bovine serum (FBS) (AusGeneX, CellMax, Australia). For serum starvation, cells were washed twice with DMEM and cultured in serum-free DMEM for 48 h before they were prepared for the subsequent experiments. The vectors including those of Human SPINK1-overexpressing, pilenti-siRNA-GFP, human shRNA against SPINK1 gene and 2nd Generation Packaging Mix were purchased from (Applied Biological Materials, Beijing, China), pCDH-CMV-ATG5-EF-copGFP (T2A) Lentivrial vector (FITGENE Biotechnology Co., Guangzhou, China), the restriction endonucleases (England Biolabs, Shanghai, China), LC3-GFP plasmid (Addgene Inc., Cambridge, MA, USA), The following commercially available antibodies were used: anti-SPINK1 (Abnova, Taiwan, China), anti-Beclin 1 (Sangon Biotech, Shanghai, China), anti-LC3 (MBL, Beijing, China), anti-p-RPS6 (Sangon Biotech, Shanghai, China), anti-p-4EBP1 (Santa Cruz, USA), anti-ATG5 (Sangon Biotech, Shanghai, China), GAPDH (Sangon Biotech, Shanghai, China). The chemicals or reagents used in this study were as follows: Lipo2000 (Invitrogen, Carlsbad, CA, USA), Cell Counting Kit-8 (Enogene Biotech, Nanjing, China), chloroquine (CQ, Sigma, St. Louis, MI, USA), 3-Methyladenine (3-MA, MedChemExpress, New Jersey, USA), puromycin (Invitrogen, USA), Gemsa Stain solution (Solarbio, Beijing, China).

### Construction of piLenti-SPINK1-shRNA-GFP and piLenti-SPINK1-overexpressing-GFP vectors

**Figure 1:**
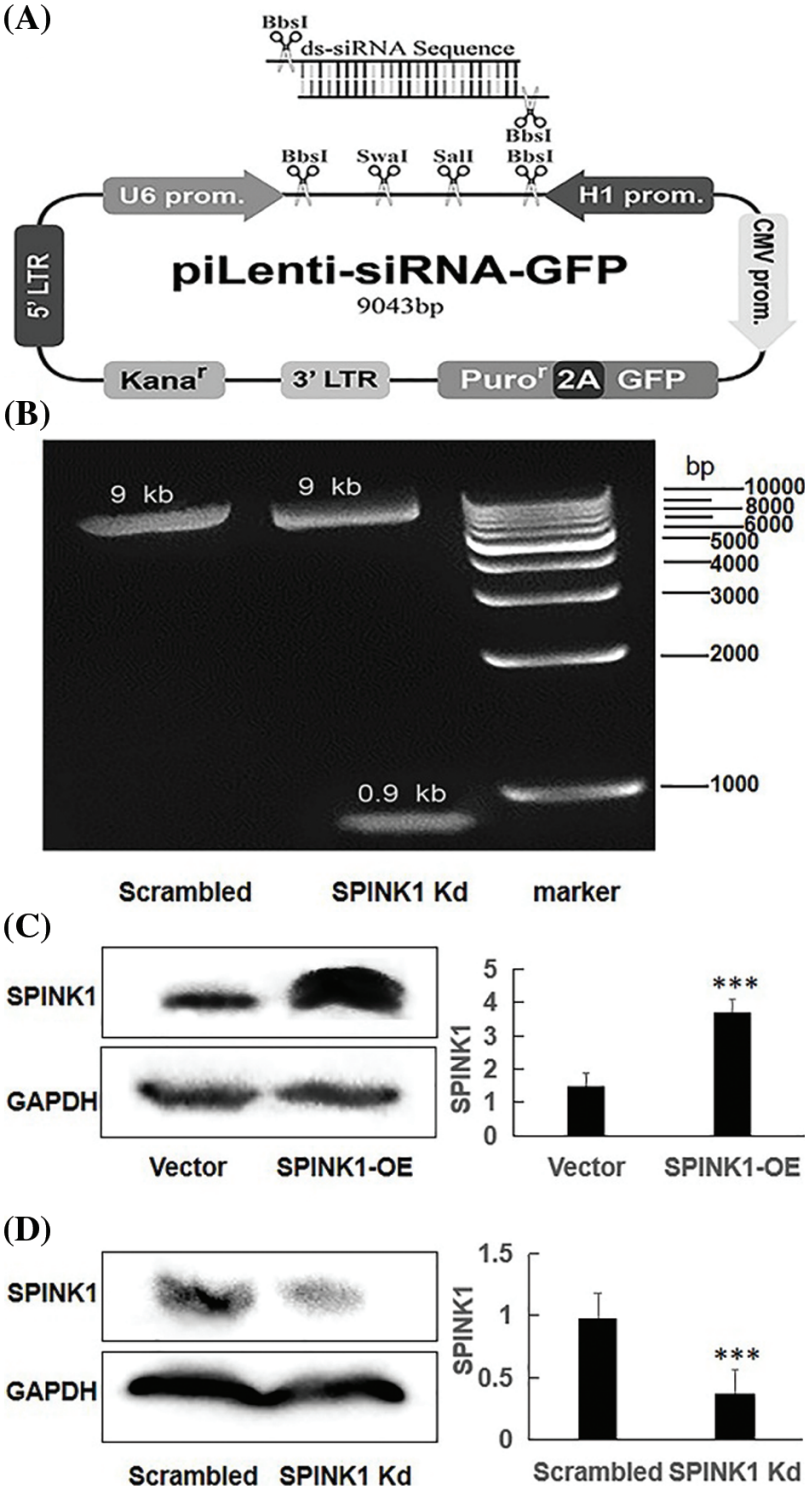
The generation of human piLenti-SPINK1-siRNA-GFP vector and permanent SPINK1 overexpression (OE) and knockdown (Kd) human HT29 cell lines. (A) Map of constructed lentiviral SPINK1-siRNA-GFP vector and location of inserted ds-siRNA sequence. (B) 0.9 kb band obtained from KpnI restriction enzyme digestion in agarose gel electrophoresis for SPINK1-siRNA lentiviral vector. The protein level of SPINK1 was tested when SPINK1 gene overexpression. (C) Verification of SPINK1 overexpression in stable SPINK1-OE HT29 cell line. (D) Identification of SPINK1 knockdown in permanent SPINK1-Kd HT29 cell line using Western blotting. Data from right respective bar charts are derived from ImageJ software and shown as mean ± SD; *p < 0.05, **p < 0.01, ***p < 0.001.

In short, the SPINK1-siRNA sequence, 5′CCAAGAUAUAUGACCCUGUTT-3′ inserted into piLenti-siRNA-GFP vector, was selected from the most efficient one among the three SPINK1 knockdown sequences reported in a previously published article [10]. TTTTTT stop signal and KpnI restriction enzymatic sequences were added to the 3′ of the above silencing sequence, while AAAA and AAAG were added to 5′ of sense and antisense strands, for the purpose of ligating with the cutting site formed by BbsI enzymatic digestion (Sense:5′—AAAACCAAGATATATGACCCTGTTTTTTTTTGGTACC—3′; Antisense:5′—AAAGGGTACCAAAAAAAAACAGGGTCATATATCTTGG—3′) (Fig. 1A). The sequence for control empty vector 5′–GGGTGAACTCACGTCAGAA—3′, and design for the control vector was all the same with SPINK1-siRNA lentiviral construct except with no KpnI insertion. All of the aforementioned sequences were synthesized by Abm. The generation of piLenti-SPINK1-overexpressing-GFP vector (piLenti-CMV-2A-GFP) was clearly described in Methods section of a published paper [11].

### Generation of stable SPINK1 overexpressing and silencing HT29 cell lines

The piLenti-SPINK1-overexpressing-GFP (piLenti-CMV-2A-GFP) and pLenti-CMV-hSPINK1-shRNA-2A-GFP vectors were transfected with either abm’s Second Generation (LV003, abm, Canada) Packaging Mix or the mixture of pMD2.G and psPAX2 packaging plasmids (Addgene Inc, Cambridge, MA, USA) into HEK-293T cells, respectively, while the transfected scrambled or empty vectors were packed simultaneously as a control. The packaged lentiviral particles were collected 24–48 h and filtered through 0.22 µm filter after the transfection. The HT29 cells were transduced by the viral particles, and those infected cells were screened under the pressure of 2 µg/ml puromycin for about 3 weeks before the puro-resistant cells were harvested [11]. Finally, the protein expression of SPINK1 was examined in the passaged HT29 cells using Western blotting.

### Measurement of proliferative capability of the cells

The puro-resistant HT29 cells that showed the significantly elevated level of SPINK1 protein were used to measure the metabolic and proliferative capability using CCK-8 kit. Seeded cells suspension (4 × 10^3^/well) in triplicates in a 96-well plate, preincubated the plate in a humidified incubator at 37°C with 5% CO_2_ for 48–72 h before adding 10 µl CCK-8 solution to each well of the plate. Then, continued to do the incubation for 4 h and measured the optical density at 450 nm using a microplate reader (Becton, Dickinson and Company, USA).

### Colony formation assay

The corresponding cell suspensions were put at 2000/well in triplicate in 6-well plates and continuously grew into colonies within 15 days. The 10% FBS culture medium changed every 5 days until the colonies appeared. Then, the plate was fixed with methanol after phosphate buffered saline (PBS) washing. Followed by three times PBS washing, the plate was stained with crystal violet (0.5% w/v). The clone formation rate was calculated using the equation: Number of clone formed in each well/the number of the cells seeded (%). Each experiment was performed three times and, finally, the data were statistically analyzed.

### Fluorescence microscope examination of GFP-LC3 punctate cells

SPINK1 overexpressing or silenced HT29 cells was transfected with pEX-GFP-hLC3WT (Addgene plasmid 24987) and pEX-GFP-hLC3ΔG (Addgene plasmid 24988) plasmids, and these cells were cultured in a 6-well plate. Then cells were incubated with complete media or serum starvation media for 48 h before they were observed under fluorescence microscope (Olympus, Tokyo, Japan).

### Western blotting

The total proteins were extracted from culturing cells using lysis buffer. Loaded 20–60 µg equal amount of protein into the wells of the SDS-PAGE gel. Run the gel for 1 h and half at 100 V, and the trans-membrane was transferred for 90 min at 120 V and blocked in 5% milk/Tris-buffered saline (TBS)/Tween (Beyotime, Shanghai, China) at room temperature for 2 h. Then the membrane was incubated with 10 µg SPINK1 (Abnova, Taiwan, China), LC3, ATG5, P-RPS6, P-4EBP1, Beclin1 (CST, Boston, MA, USA) or GAPDH primary antibody (Sangon Biotech, Shanghai, China) at 1:1000 dilution overnight before washing with 5% milk/TBS/Tween. The secondary antibody (Abcam, Cambridge, UK) at 1:5000 dilution was mixed in 5% milk/TBS/Tween for 1 h, followed by three times washing. Add 500 µl A and B developer (Tanon, Shanghai, China) on the membrane, respectively, for 5 min and developed using Gel Image Lab System (ChemiDoc + Gelimaging System, BIO-RAD, USA). The developed band density was evaluated using Gelpro32 image analyzing software.

### Statistical analysis

The mean and standard deviation (mean ± SD) were calculated for each item in the experiments. The data were collected from independent experiments or experiments run in triplicates. A Student’s *t*-test was used to analyze the significant difference between experimental groups, and *p*-values of < 0.05, < 0.01 and < 0.001 were considered statistically significant (*), more significant (**) and highly significant (***), respectively.

## Results

### Construction of SPINK1-shRNA lentiviral vector

**Figure S1:**
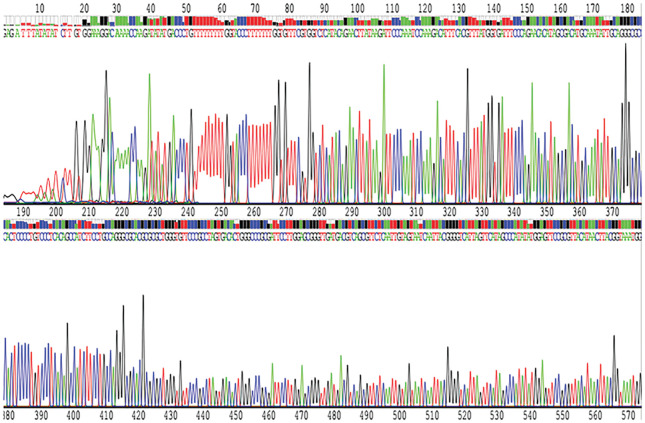
The map showed that the recombinant lentiviral vector inserted with shRNA fragment against SPINK1 gene was finally confirmed by DNA sequencing.

The map of a recombinant lentiviral vector inserted with shRNA fragment against SPINK1 gene was showed in Fig. 1A, and the constructed vector was finally confirmed by DNA sequencing (Suppl. Fig. 1), followed by Western blotting that was carried out to approve the downregulation of SPINK1 expression after transfection of the lentiviral-vector into HT29 cells. The agarose gel electrophoresis showed a clearly expected band after digestion with kpnI digestion enzyme for the vector (Fig. 1B). The tagged fluorescent GFP was observed in the 80% of HT29 cells after transfection (data not shown). Compared with the scrambled control, the immunoblot exhibited decreased SPINK1 expression after the transfection of HT29 cells with SPINK1-shRNA construct, suggesting the successful generation of SPINK1-knockdown lenti-vector (Fig. 1D).

### Establishment of permanent SPINK1-OE and SPINK1-KD HT29 cell lines

The successful setup of the permanent SPINK1-OE and SPINK1-Kd HT29 cell lines were certified by Western blotting, showing either the high or low expression of SPINK1 corresponding to the overexpressing and knockdown of the respective cells, respectively (Figs. 1C, 1D).

### Tumor cell proliferation and clonogenic survival via ectopic SPINK1 expression

**Figure 2:**
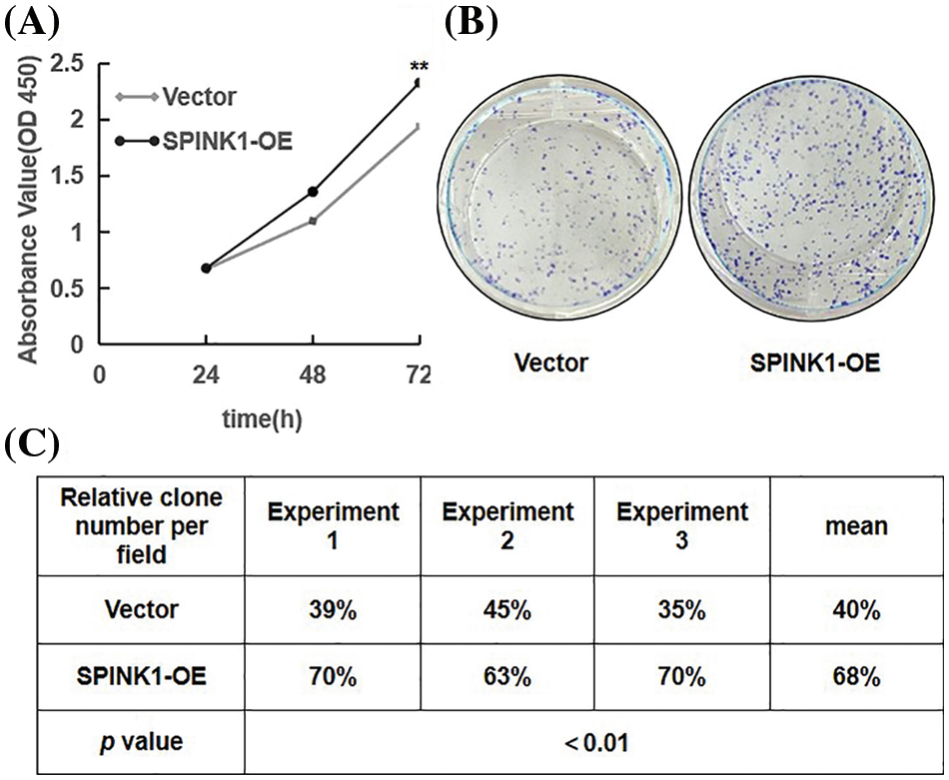
Proliferation and colony formation in stably SPINK1-OE HT29 cells. (A) Line chart showing proliferation capability measured using CCK-8 assay at varied time points. (B) Image for colony formation from SPINK1-OE HT29 cells on surface of petri dishes after GIEMSA staining. (C) Table showing number of colonies formed from the above SPINK1-OE HT29 cells in repeated experiments. Data are shown as mean ± SD; **p < 0.01.

Prior to explore the possible signaling pathway associated with SPINK1’s function, we evaluated the effects of SPINK1 on the growth of colorectal tumor HT29 cells. We initially generated stable HT29 cell lines lentiviral overexpressed or silenced human SPINK1 gene before carrying out CCK-8 and clonogenic formation assays to assess the proliferative and clonal-survival impact of SPINK1 on these cells. Compared with the empty-vector control, the metabolic and proliferative capability measured by CCK-8 assay increased significantly in SPINK1-OE HT29 cells at varied time points, 24, 48 and 72 h after the seeding (Fig. 2A, *p* < 0.01). The clonogenic assay showed that the number of colonies formed in SPINK1-OE HT29 cells was greater than mock-vector control (Fig. 2B). The mean value of the number of colonies from three independent experiments was 68%, compared with an average of 40% from the three corresponding controls (Fig. 2C, *p* < 0.01). Altogether, these data suggests that SPINK1 possessed the ability to promote the proliferation and clonogenesis of HT29 cells.

### SPINK1 induced enhancement of autophagy in HT29 cells

**Figure 3:**
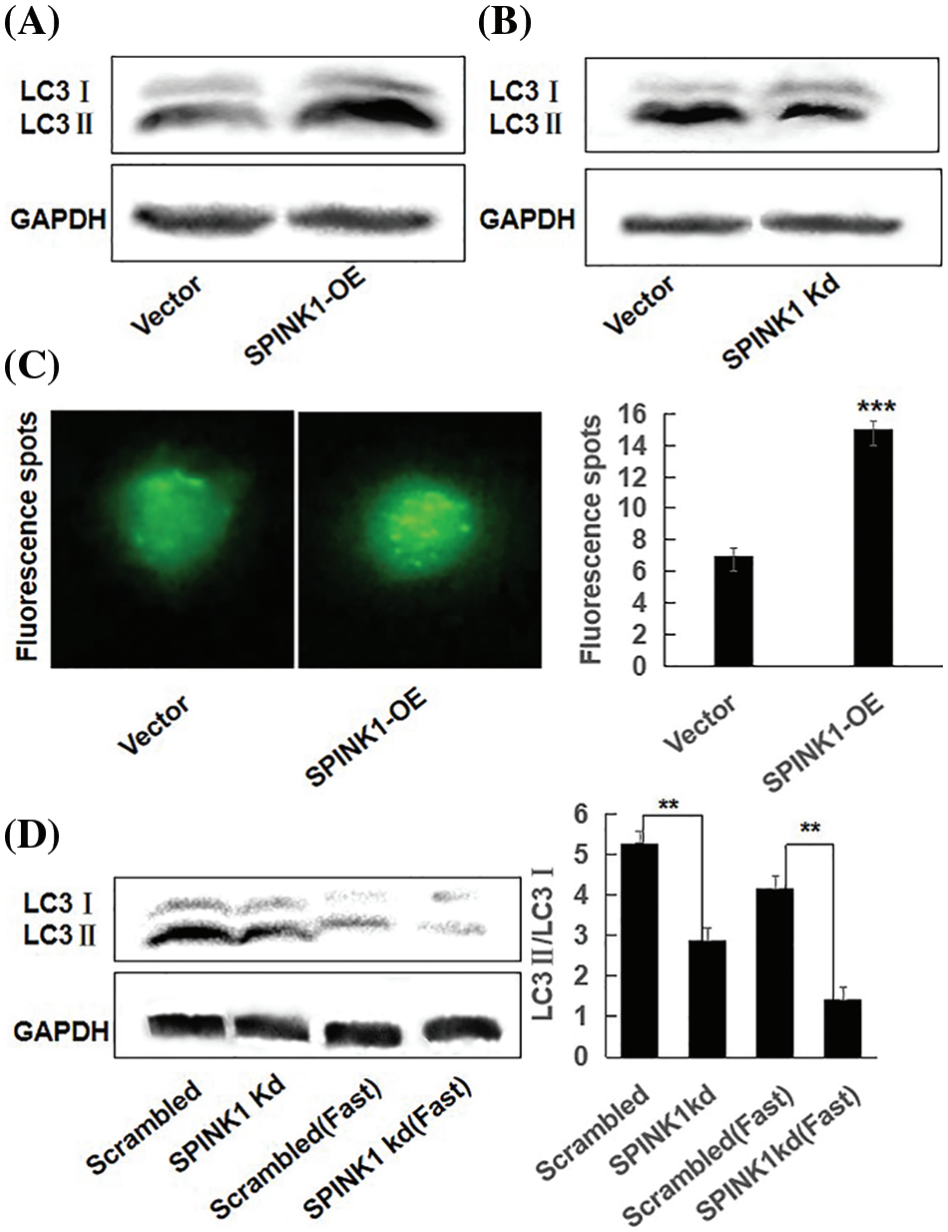
SPINK1 regulated autophagy in HT29 cells; (A) SPINK1-induced enhancement of autophagy (LC3) in stable SPINK1-OE HT29 cell line using Western blotting. (B) Amelioration of autophagy (LC3) measured by Western blotting in permanent SPINK1-Kd HT29 cells. (C) Green fluorescent LC3 punctuate seen under fluorescent microscope and corresponding histogram post-transfection of plasmid in SPINK1-OE HT29 cells. Scale bar = 25 μm. (D) Detection of LC3 protein level using Western blotting in stable SPINK1-Kd HT29 cells under normal and fasting conditions, followed by transformed bar graph on right side. Data are shown as mean ± SD; **p < 0.01, ***p < 0.001.

As the loss of SPINK1 gene resulted in enhanced autophagy in normal pancreatic cells of the mice [5], we hypothesized that autophagy may participate in the process of SPINK1-induced growth of the cells. To verify whether SPINK1-stimulated HT29 cell’s proliferation was associated with autophagic event, we generated two types of lentiviral transduced HT29 cell lines which permanently overexpressing wild type SPINK1 or silencing SPINK1, and measured their autophagy level under the conditions of normal culturing and starvation cultivation using Western blotting. Notably, compared to empty-vector control, SPINK1-OE HT29 cells cultured in normal medium displayed an elevated amount of LC3II and a raised ratio of LC3II/LC3I, indicating the marked induction of autophagic event (Fig. 3A). In contrast, SPINK1 Kd HT29 cells in normal culturing medium showed lower ratio of LC3II/LC3I (Fig. 3B), suggesting the occurrence of ameliorated autophagy state under this circumstance. Consistent with the above findings, a more punctuate distribution pattern was exhibited under a fluorescence microscope 48 h post-transfection of pEX-GFP-hLC3WT plasmid into SPINK1-OE HT29 cells, in comparison with pEX-GFP-hLC3ΔG vector control, cultured under normal condition (Fig. 3C, *p* < 0.001). Under overnight fasting condition, SPINK1-Kd HT29 cells significantly lowered the ratio of LC3II/LC3I in comparison with those HT29 cells transfected with scrambled vector (Fig. 3D, *p* < 0.001). Taken together, SPINK1 regulated the expression ratio of LC3II/LC3I, a critical parameter for evaluation of autophagy in HT29 cells.

**Figure S2:**
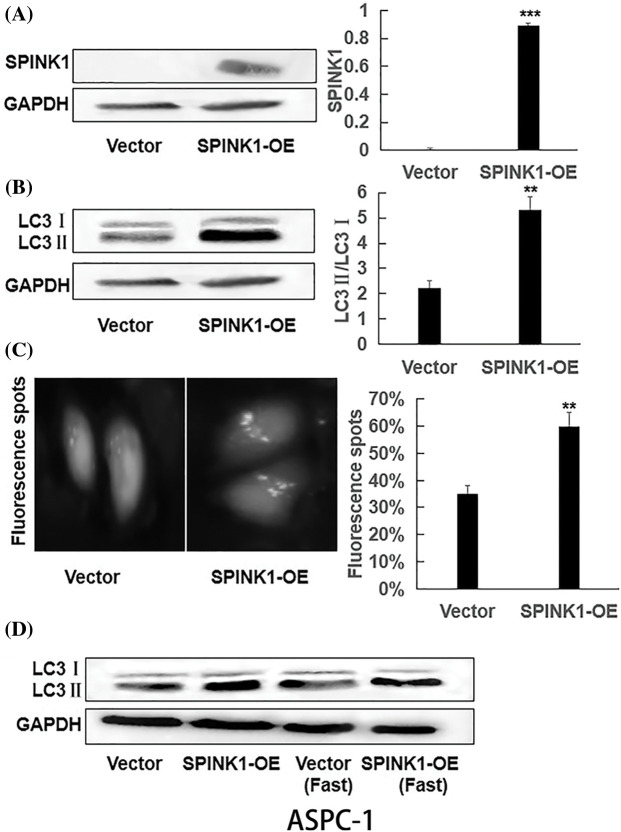
The generation of permanent SPINK1-OE human AsPC-1 cell line and SPINK1-regulated autophagy in AsPC-1 cells; (A) Western blotting analysis of SPINK1 overexpression in lentivirally-transduced SPINK1-OE AsPC-1 cell line. (B) Verification of LC3II upregulation in stable SPINK1-OE AsPC-1 cell line using Western blotting. (C) Green fluorescent LC3 punctuate seen under fluorescent microscope and corresponding histogram post-transfection of plasmid in stable SPINK1-OE AsPC-1 cells. Scale bar = 25 μm. (D) Detection of LC3 protein level using Western blotting in stable SPINK1-OE AsPC-1 cells under normal and fasting conditions. Data are shown as mean ± SD; **p < 0.01, ***p < 0.001.

Simultaneously, we tested the generality of the above findings in pancreatic carcinoma AsPC-1 cells, the results showed that SPINK1-OE lentiviral vector-transduced AsPC-1 cells displayed a higher expression of SPINK1 protein (Suppl. Fig. 2A); that displayed the higher ratio of LC3II/LC3I (Suppl. Fig. 2B), compared with empty-vector control cells which was consistent to more GFP-tagged LC3 green puncta under a fluorescence microscope (Suppl. Fig. 2C); that also revealed a higher ratio LC3II/LC3I under cellular fasting condition, compared to the starved control cells (Suppl. Fig. 2D).

**Figure 4:**
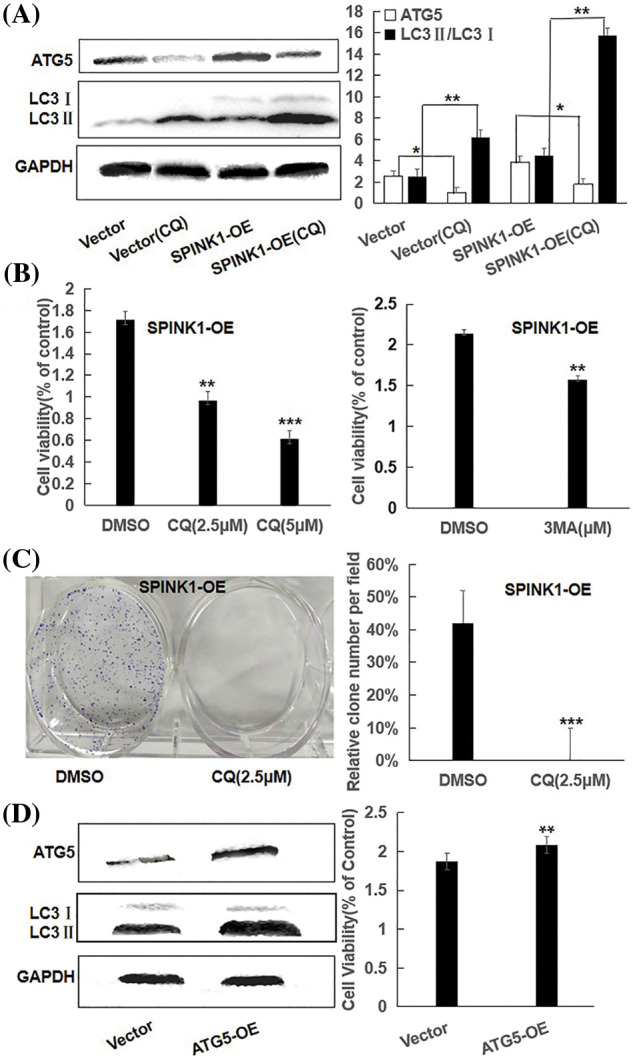
Proliferation capability and clonogenic cell survival via autophagic regulation in SPINK1-OE HT29 cells; (A) Reduction of ATG5 and LC3 in parental and SPINK1-OE HT29 cells by CQ, autophagy inhibitor, using Western blot, along with respective bar chart. (B) CCK-8 measurement of proliferation capability in SPINK1-OE HT29 cells treated by the autophagy inhibitor CQ and 3-MA for 36 h, respectively. (C) Colony formation assay and column graph for SPINK1-OE HT29 cells treated with autophagy inhibitor CQ for 14 days, followed with crystal violet staining of attached cells. (D) CCK-8 measurement of proliferation capability (bar graph) of SPINK1-OE HT29 cells by upregulating autophagy upon overexpression of transfected ATG5 vector, shown by Western blot. Data are shown as mean ± SD; *p < 0.05, **p < 0.01, ***p < 0.001.

It was plausible that the GFP-LC3 puncta accumulation or the increased LC3II expression could result from either an increment in autophagy activity or a block in the later stages of the autophagic process, such as impaired autophagosome degradation [12]. To roughly discriminate between these two possible events, we examined the alterations of LC3II/LC3I ratio in empty vector and SPINK1 transduced HT29 cells, respectively, after the treatment of chloroquine (CQ), a blocker of lysosomal acidification and subsequent autophagosome degradation [13]. As compared to ‘empty vector’ control cells with or without CQ addition, the inhibition of autophagosome degradation by CQ significantly increased LC3-II/LC3I conversion in both SPINK1-OE and empty-vector control HT29 cells, suggesting the autophagic activity was possibly enhanced once SPINK1 gene was amplified in these cells (Fig. 4A, *p* < 0.01). Moreover, the expression of ATG5, one of the essential molecules for autophagic pathway [14], was remarkably elevated in the above SPINK1-OE cells and even upgraded in extent with CQ addition, again confirmed the function of SPINK1 in autophagy enhancement in this regard (Fig. 4A). The above data indicated the presence of reinforced autophagic event was closely associated with SPINK1 abundance in HT29 cells.

### Inhibition and enhancement of clonogenic survival and/or cell proliferation by autophagy regulation

To determine whether autophagy induction itself was closely associated with the proliferation and clonogenic formation of HT29 cells, we initially used CQ to inhibit enhanced autophagy caused by SPINK1 upregulation. Expectedly, CQ increased the amount of LC3II and significantly blocked the growth and clonal formation of SPINK1-OE HT29 cells (Figs. 4B, 4C), suggesting that the downregulation of autophagy activity could influence the HT29 cell growth in reverse direction. To further consolidate this finding, we next used 3-MA, another selective autophagy suppressor as described above, to block the autophagy in the initial steps of the process. The result showed similarly decreased cell proliferation by 3-MA treatment in the aforementioned cell lines (Fig. 4B). Then we infected the HT29 cells with Atg5 overexpressing lentiviral vector to upregulate the expression of autophagy protein Atg5, a required and essential component in the formation of Atg5/Atg7 dependent autophagy [15]. Compared with control, the immunoblotting showed that Atg5 and LC3II/LC3I conversion were significantly increased after Atg5 gene transfer, and the upregulation of Atg5 significantly enhanced cell growth as demonstrated by the presence of increased cellular viability in the above indicated HT29 cells (Fig. 4D). Altogether, autophagy regulation was closely linked to HT29 cell viability, and in other words, HT29 cell’s growth and survival were autophagy-dependent, at least to some extent.

### Independence of mTOR, and association of SPINK1 induced autophagy with Beclin 1 regulation

**Figure 5:**
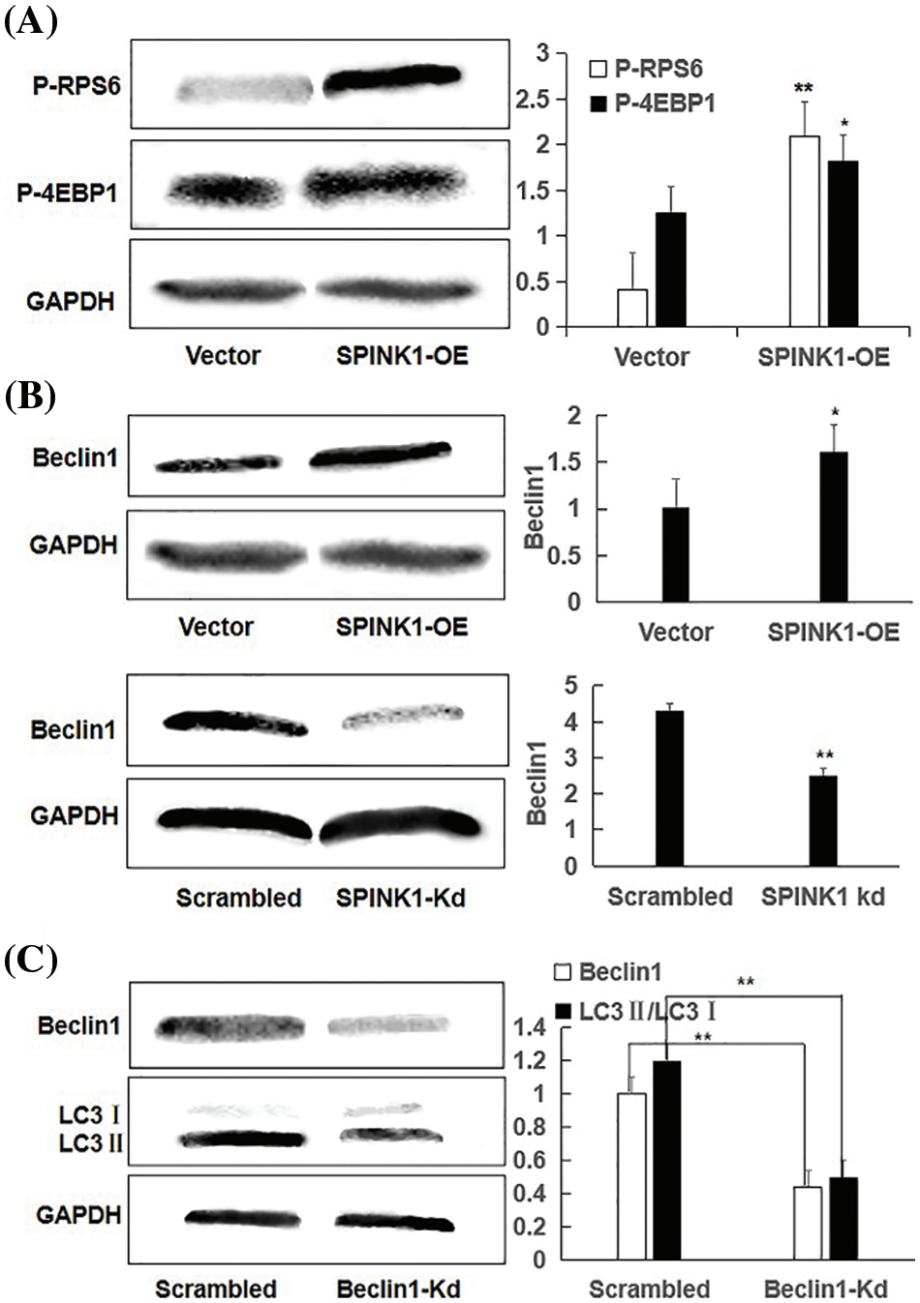
Independent of mTOR, Beclin1-regulated enhanced autophagy using representative Western blotting in SPINK1-OE and SPINK1-Kd HT29 cells, relative to their corresponding controls; (A) Detection of activation of phospho-RPS6 (p-RPS6) and phospho-4EBP1 (p-4EBP1) in stable SPINK1-OE HT29 cells using Western blotting. (B) Higher level expression of Beclin1 in permanent SPINK1-OE HT29 cells using Western blotting. (C) Western blotting for decreased Beclin1 expression in stable SPINK1-Kd HT29 cells. (D) Western blot analyses of LC3I/LC3II ratio in HT29 cells tranfected with Beclin1-shRNA vector. Data are shown as mean ± SD; *p < 0.05, **p < 0.01.

**Figure S3:**
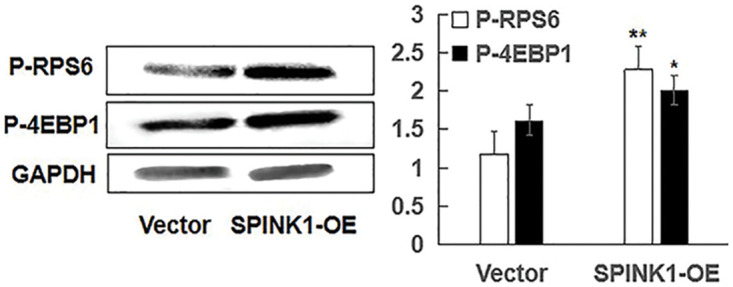
Independent of mTOR, SPINK1-induced enhanced autophagy using representative Western blotting in SPINK1-OE AsPC-1 cells; Detection of activation of phospho-RPS6 (p-RPS6) and phospho-4EBP1 (p-4EBP1) in stable SPINK1-OE AsPC-1 cells using Western blotting. Data are shown as mean ± SD; *p < 0.05, **p < 0.01.

To further evaluate whether the activated autophagy was initiated through the typical mTOR or non-mTOR signaling pathway in the SPINK1-OE HT29 cells, we measured the expression of p-4EBP1 and p-RPS6, two important downstream signaling proteins of mTOR, a crucial route in coordinating cell growth with growth-related factors or cell’s microenvironment [16]. The expression levels of p-4EBP1 and p-RPS6 were clearly increased in SPINK1-OE HT29 cells (Fig. 5A, *p* < 0.05, *p* < 0.01), suggesting that the mTOR pathway-induced autophagy was unlikely owing to the activation of this molecular route that usually inhibits autophagy [17]. Namely, this finding was exactly contradicted to the enhanced autophagy observed in the above SPINK1-OE cells. In other words, SPINK1-stimulated enhancement of autophagy in HT29 cells was not mediated via mTOR signaling, implying the activation of a disparate autophagy signaling in this scenario. To expand the generality of this discovery, we tested the above-mentioned, stable SPINK1-OE AsPC-1 cell line, selected AsPC-1, another pancreatic tumor cell line with glandular tissue origin, and the results showed that the expression of p-4EBP1 and p-RPS6 were significantly increased compared with the empty-vector control cells, suggesting the similar mTOR-independent pathway for SPINK1-induecd autophagy in AsPC-1 cells (Suppl. Fig. 3). In summary, SPINK1 overexpression remarkably enhanced basal and starvation-induced autophagy in HT29 cells, and this process could be implemented probably through a mTOR-independent mechanism.

To explore the possible non-mTOR signaling pathway that SPINK1 may trigger in this case, we next checked another autophagy-related protein Beclin1, another critical regulator of the autophagic pathway [18]. To be exact, we examined whether ‘oncogenic’ SPINK1 could lead to the altered expression of Beclin1, independently of mTOR signaling. For this purpose, we used permanent SPINK1-OE or silencing HT29 cells to upregulate or downregulate SPINK1 expression. As noted in Fig. 5B, an obvious increase in Beclin1 protein level could be observed in SPINK1-OE HT29 cells, whereas a marked decrease in Beclin-1 amount could be revealed in SPINK1 knockdown HT29 cells, compared to mock-vector and scrambled controls. Collectively, these data indicated that SPINK1 induced-autophagy enhancement in HT29 cells of colorectal carcinoma was likely accomplished via Beclin1 signaling.

Then we speculated that SPINK1 induction of autophagy was expected to be linked to Beclin1 regulation. To approve this assumption, we used the previously validated lentiviral shRNA-Beclin1 construct to downregulate Beclin1 expression in SPINK1-OE HT29 cells. Notably, the transfer of Beclin1 shRNA vector significantly decreased LC3II/LC3I ratio in these cells (Fig. 5C), suggesting the role of Beclin1 in the regulation of SPINK1-induced autophagy. In summary, SPINK1-deduced increased autophagy event was closely associated with the downregulation of Beclin1 in HT29 cells.

## Discussion

The signaling pathway of SPINK1 (TATI) has long been obscure in the growth stimulation of CRC cells [1–3,10]. In this study, we provided some new data to preliminarily demonstrate the presence of an autophagy-linked signaling route which was closely associated with SPINK1-induced proliferation of HT29 cells. Moreover, SPINK1-induced autophagy was closely associated with Beclin1 regulation. This possibly pave a new avenue for understanding the molecular mechanisms which is likely responsible the roles of SPINK1 in the pathogenesis of CRC, even in other types of cancers.

Though SPINK1 has been known to be a negative regulator of autophagy in normal murine pancreatic cells [5], the connection between SPINK1 and autophagy remains unknown on different cancer cells. We likely for the first time disclosed the ‘mystery’ that SPINK1 could enhance autophagy in HT29 cells, instead of ameliorating this event as happened in the pancreatic acinar cells of the normal mouse [5]. This interesting finding exhibited the dual and opposite roles of SPINK1 in both normal and cancerous cells, and emphasized the possibility that the same molecule was able to show the ‘two sites of a coin’ under different physiological and pathological conditions, even though we were not aware of the underlying principle. The pro-growth and anti-survival functions of autophagy could be observed in colorectal cancer, although the mechanisms downstream of autophagy (to reduce or enhance tumor growth) were not well known [19–21]. In the context of SPINK1, our data favored Kon M’s report in which it was described that the chaperone-mediated autophagy was required for colon tumor growth [22], showing a new signaling route of SPINK1 that promoted colon cancer growth via reinforced autophagy.

In this study, we showed a new Beclin1-associated autophagic pathway which had involved in SPINK1-induced proliferation of HT29 cells, and this autophagy-related, possible signaling of the action in colorectal cancer development have not yet been unraveled. Although SPINK1 was reported to be likely acting via epidermal growth factor receptor (EGFR) pathway in several publications [23,24], there still lacked some consolidated evidence to support that SPINK1-induced autophagy was carried out through EGFR route, as Yamamuya’s data demonstrated that the autophagic activity was not raised in acinar cells of pancreas from selevtive EGFR knockout mice [8]. Likewise, Gorzalczany et al. stated that EGFR activation suppressed autophagic event and EGFR inhibitor enhanced autophagy in non-small-cell lung carcinoma (NSCLC) [25], which were contrary to our ‘enhanced autophagy’ occurred in SPINK1-OE HT29 cells. These data favor the temporary conclusion that SPINK1-induced autophagy enhancement in HT29 cells was accomplished via an EGFR-independent signaling route.

Our data further excluded the possibility of mTOR centered autophagy route in the context of SPINK1-induced HT29 cell growth, as the activation of mTOR downstream signals would have suppressed autophagy process. Independently of mTOR, Beclin1 was found to be closely associated with the regulation of SPINK1-stimulated autophagy, which more likely shed light on the discovery of a new pathway responsible for SPINK1’s function in colorectal and other types of cancers.

Beclin-1 functions as an important player in the Beclin1/VPS34 assembly comprising autophagy stimulatory and inhibitory proteins that regulate autophagosome formation [26]. As a matter of fact, a high percentage of positive immunohistochemical staining of SPINK1 was observed in colorectal cancer patients [27,28], and interestingly, Chang Hyeok Ahnalso noticed that the increased expression of Beclin-1 was detected in 95% of the tumor tissues of the CRC patients [29]. However, the possible connections and significance of respectively elevated expression of SPINK1 and Beclin1 in the specimens of CRC patients remains unclear and need to be further investigated.

In this investigation, we presented some new data with respect to the molecular pathway through which SPINK1 may simulate HT29 cell’s growth, and these new findings partially filled the gaps among SPINK1, autophagy and tumor cell’s survival, and will possibly even shed light on the discovery of the signaling route that is responsible for the proliferation, invasion and metastasis of CRC. Moreover, our study will simultaneously provide some new thoughts and hints for deep exploration of SPINK1-related molecular mechanisms involved in the pathogenesis of other types of cancers. Nevertheless, further ensuing experiments and analyses are required to be carried out both on cellular and molecular levels before we are able to reach this goal.

## Data Availability

All data are available in the manuscript.
